# Supplementary figures and images for: Ulk4 promotes Shh signaling by regulating Stk36 ciliary localization and Gli2 phosphorylation
Source: eLife. 2023 Dec 14;12:RP88637. doi: 10.7554/eLife.88637 (PMC10721220; doi:10.7554/eLife.88637)

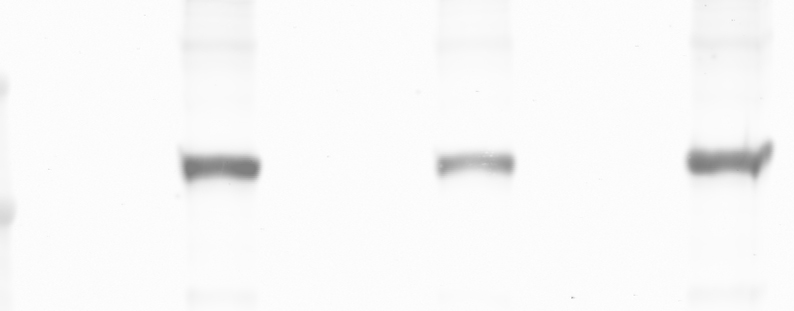

Supplement: Figure 1—source data 1. [file elife-88637-fig1-data1.zip › Figure1- source data1/Figure1E/figure1E-1.jpg]

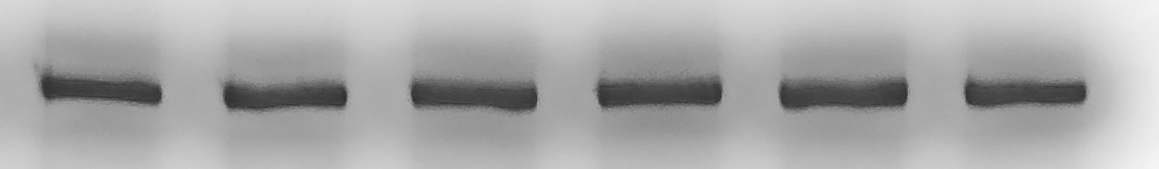

Supplement: Figure 1—source data 1. [file elife-88637-fig1-data1.zip › Figure1- source data1/Figure1E/figure1E-2.jpg]

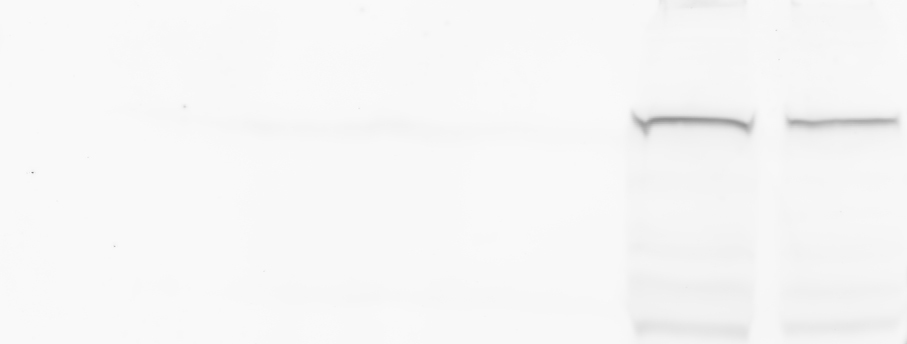

Supplement: Figure 1—source data 1. [file elife-88637-fig1-data1.zip › Figure1- source data1/Figure1E/figure1E-3.jpg]

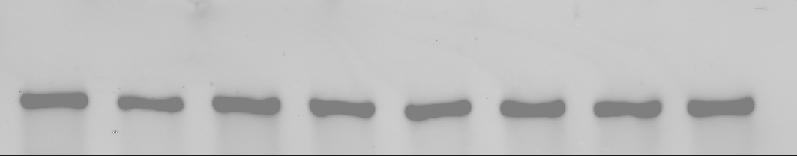

Supplement: Figure 1—source data 1. [file elife-88637-fig1-data1.zip › Figure1- source data1/Figure1F/figure1F-1.jpg]

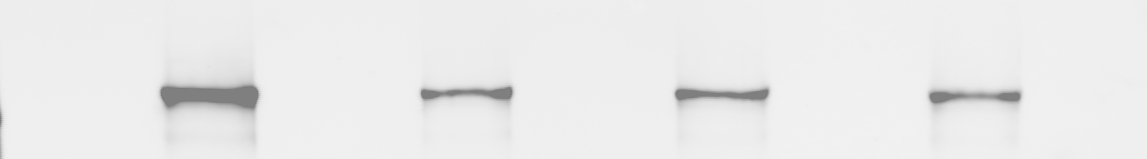

Supplement: Figure 1—source data 1. [file elife-88637-fig1-data1.zip › Figure1- source data1/Figure1F/figure1F-2.jpg]

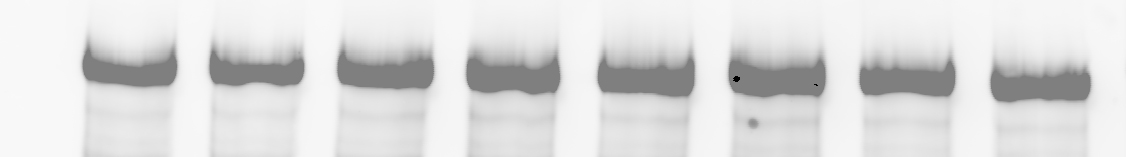

Supplement: Figure 1—source data 1. [file elife-88637-fig1-data1.zip › Figure1- source data1/Figure1G/figure1G-1.jpg]

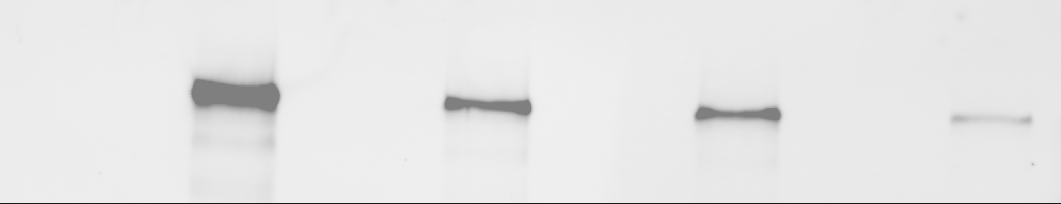

Supplement: Figure 1—source data 1. [file elife-88637-fig1-data1.zip › Figure1- source data1/Figure1G/figure1G-2.jpg]

Figure1E

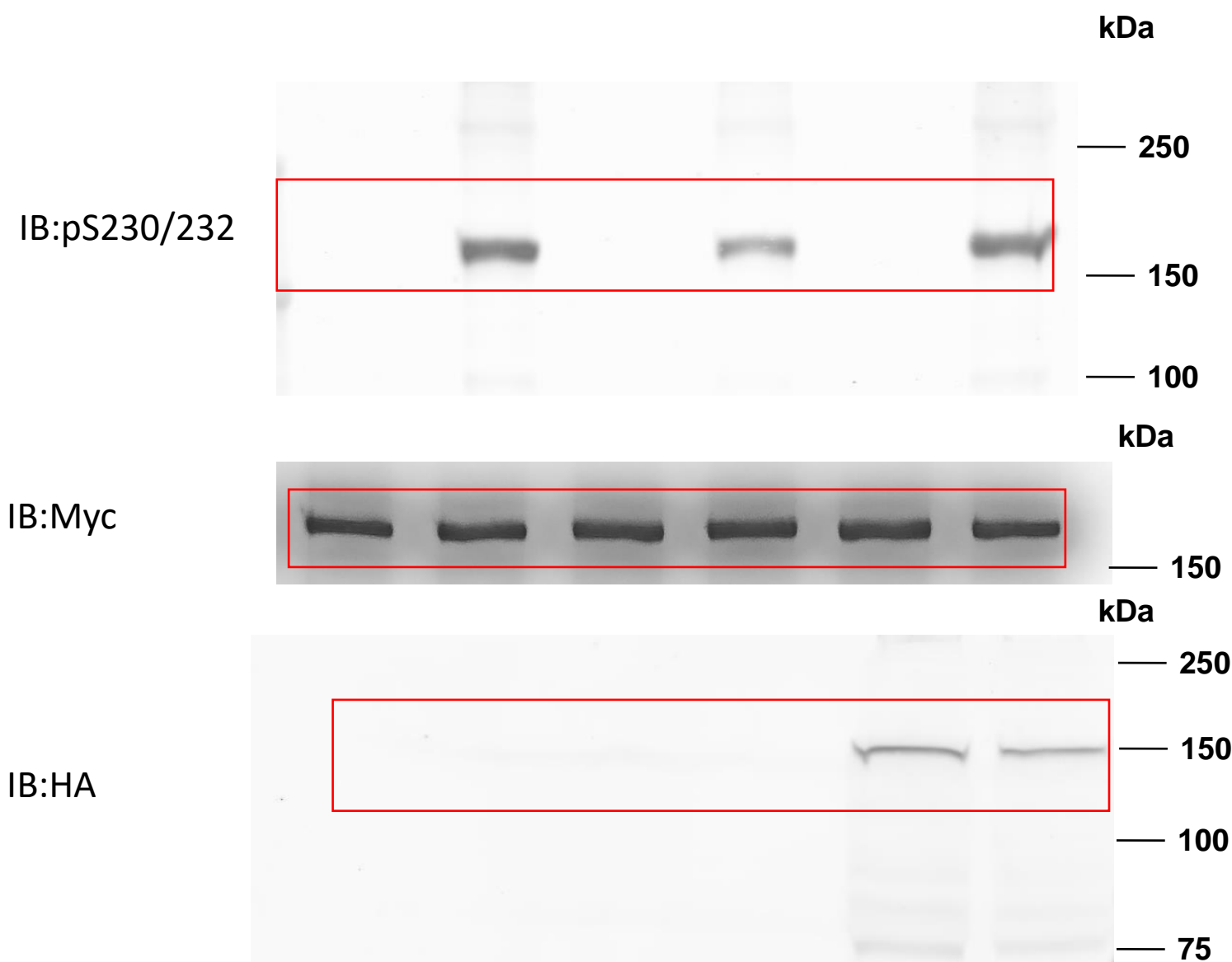

Figure1F

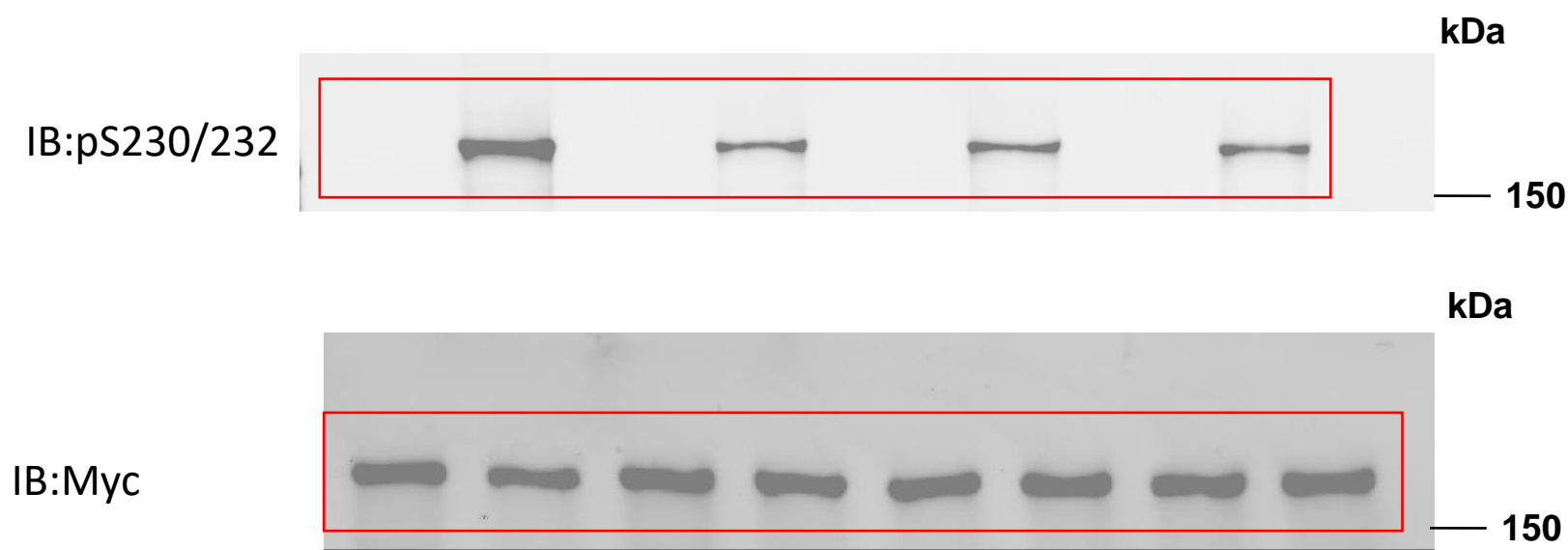

Figure1G

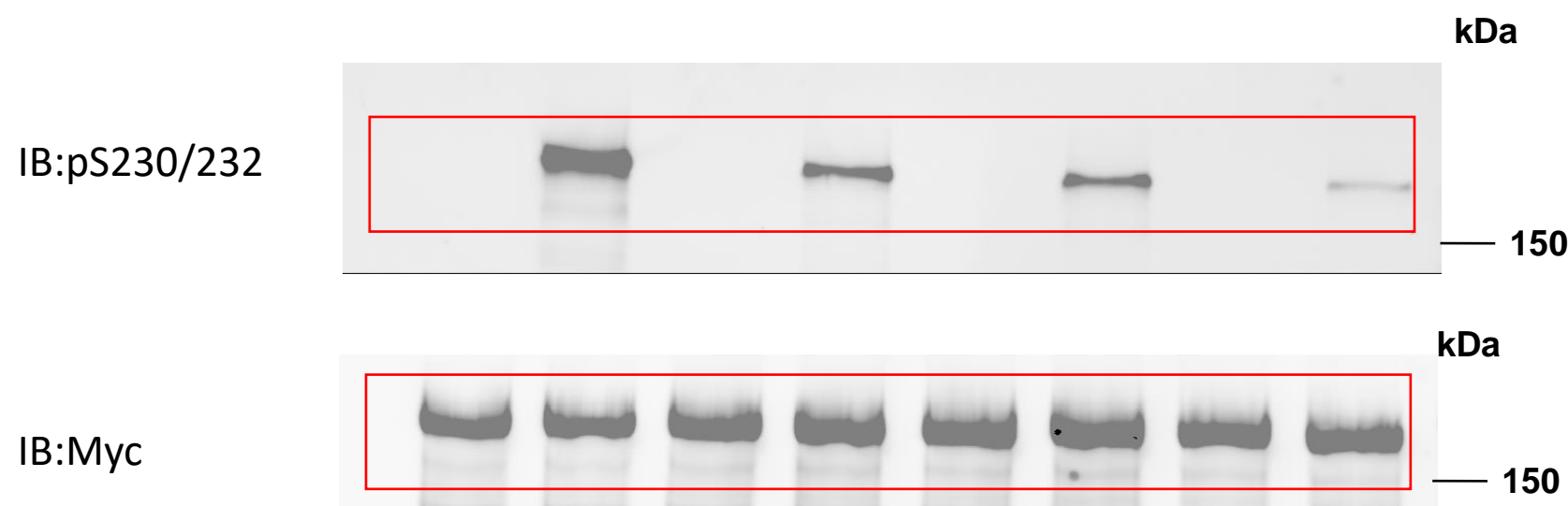

Supplement: Figure 1—source data 1. [file elife-88637-fig1-data1.zip › Figure1- source data1/Figure1-source data 1.pdf]

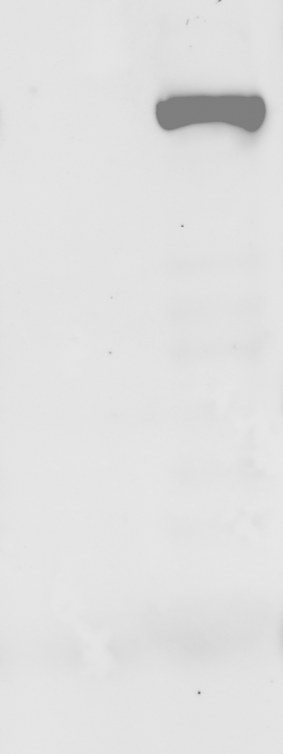

Supplement: Figure 2—source data 1. [file elife-88637-fig2-data1.zip › Figure2-source data1/Figure2A/Figure2A-1.tif]

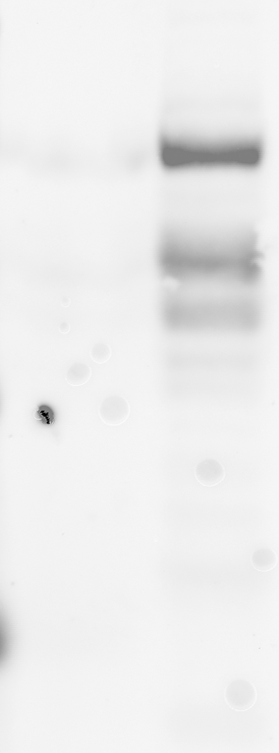

Supplement: Figure 2—source data 1. [file elife-88637-fig2-data1.zip › Figure2-source data1/Figure2A/Figure2A-2.tif]

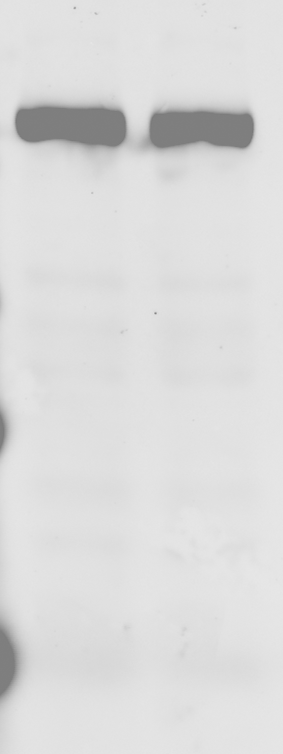

Supplement: Figure 2—source data 1. [file elife-88637-fig2-data1.zip › Figure2-source data1/Figure2A/Figure2A-3.tif]

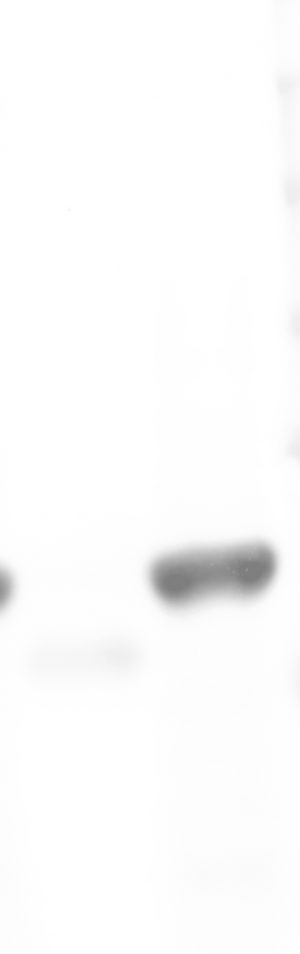

Supplement: Figure 2—source data 1. [file elife-88637-fig2-data1.zip › Figure2-source data1/Figure2B/Figure2B-1.tif]

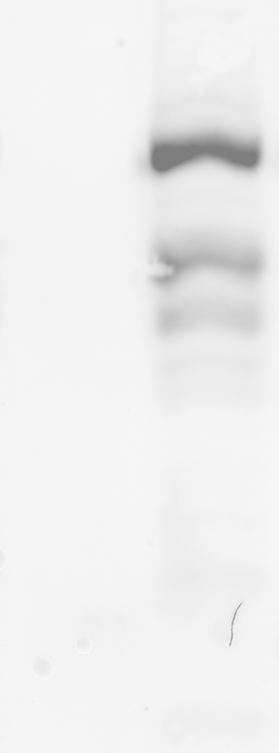

Supplement: Figure 2—source data 1. [file elife-88637-fig2-data1.zip › Figure2-source data1/Figure2B/Figure2B-2.tif]

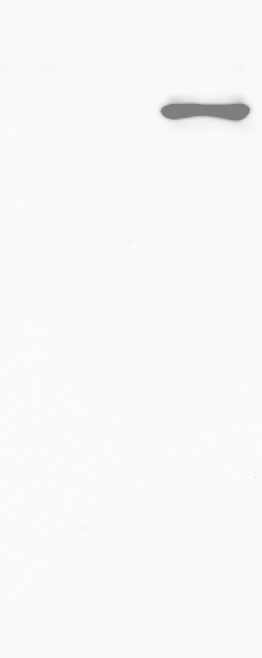

Supplement: Figure 2—source data 1. [file elife-88637-fig2-data1.zip › Figure2-source data1/Figure2B/Figure2B-3.tif]

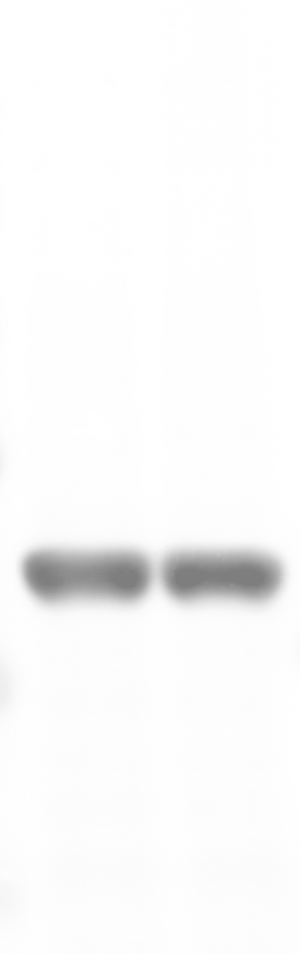

Supplement: Figure 2—source data 1. [file elife-88637-fig2-data1.zip › Figure2-source data1/Figure2B/Figure2B-4.tif]

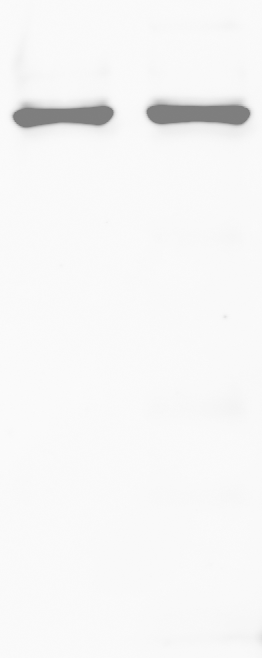

Supplement: Figure 2—source data 1. [file elife-88637-fig2-data1.zip › Figure2-source data1/Figure2B/Figure2B-5.tif]

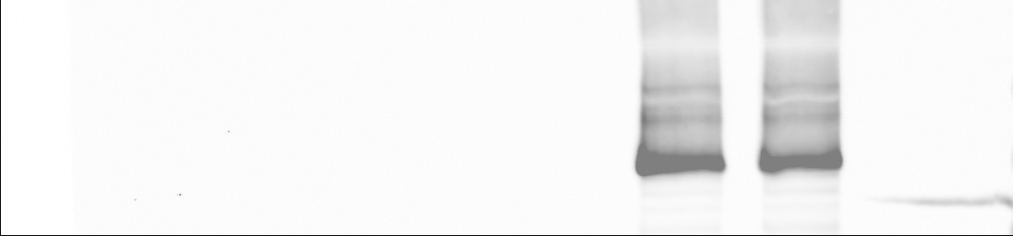

Supplement: Figure 2—source data 1. [file elife-88637-fig2-data1.zip › Figure2-source data1/Figure2D/Figure2D-1.jpg]

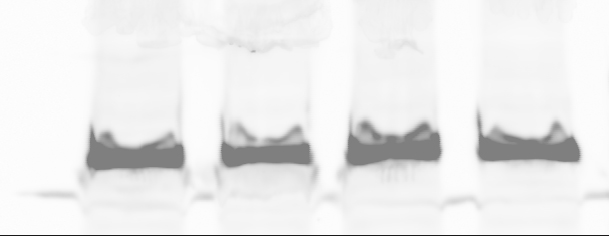

Supplement: Figure 2—source data 1. [file elife-88637-fig2-data1.zip › Figure2-source data1/Figure2D/Figure2D-2.jpg]

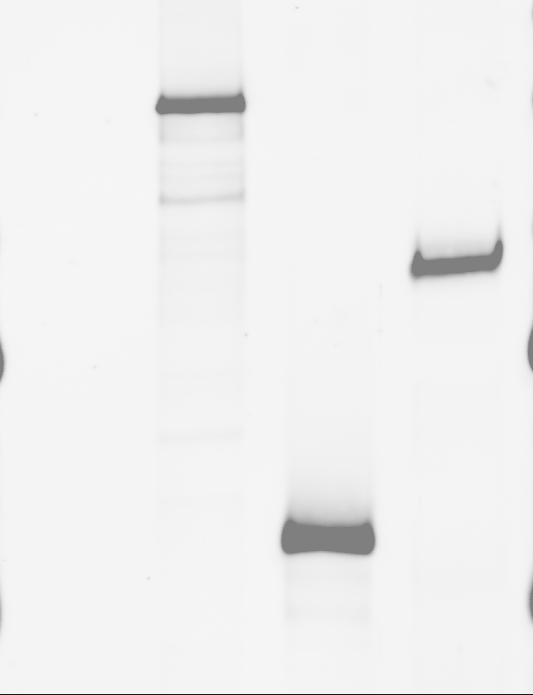

Supplement: Figure 2—source data 1. [file elife-88637-fig2-data1.zip › Figure2-source data1/Figure2D/Figure2D-3.jpg]

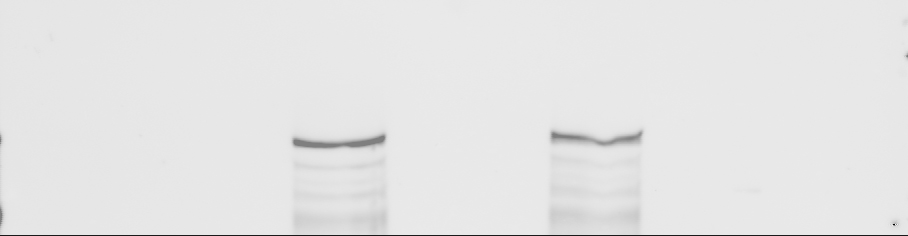

Supplement: Figure 2—source data 1. [file elife-88637-fig2-data1.zip › Figure2-source data1/Figure2E/Figure2E-1.jpg]

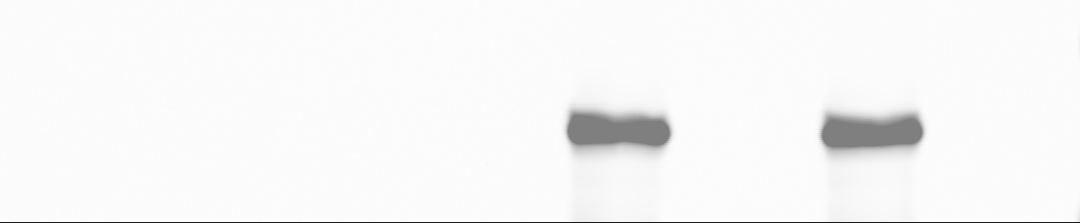

Supplement: Figure 2—source data 1. [file elife-88637-fig2-data1.zip › Figure2-source data1/Figure2E/Figure2E-2.jpg]

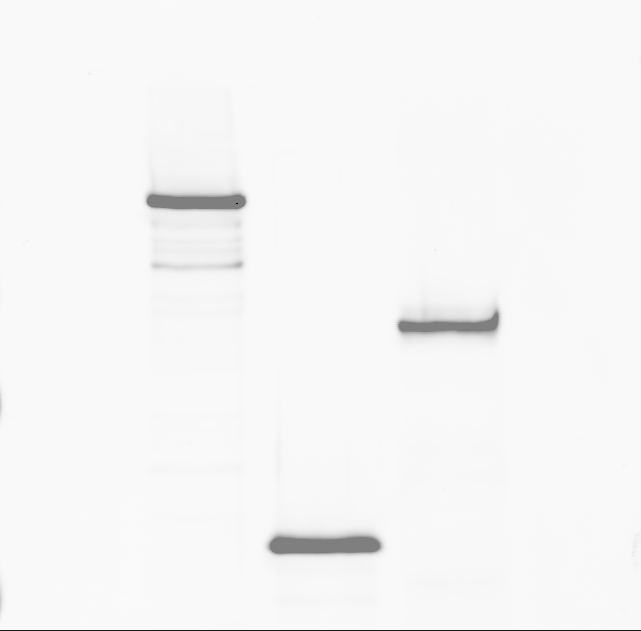

Supplement: Figure 2—source data 1. [file elife-88637-fig2-data1.zip › Figure2-source data1/Figure2E/Figure2E-3.jpg]

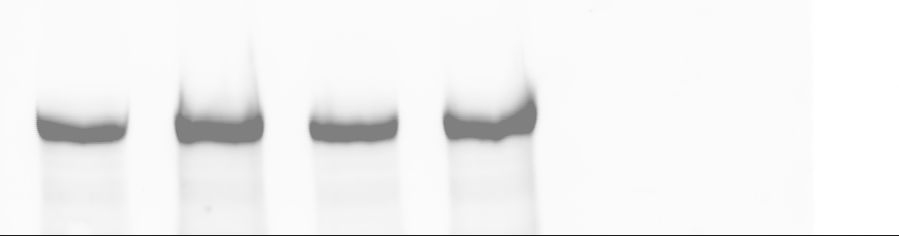

Supplement: Figure 2—source data 1. [file elife-88637-fig2-data1.zip › Figure2-source data1/Figure2E/Figure2E-4.jpg]

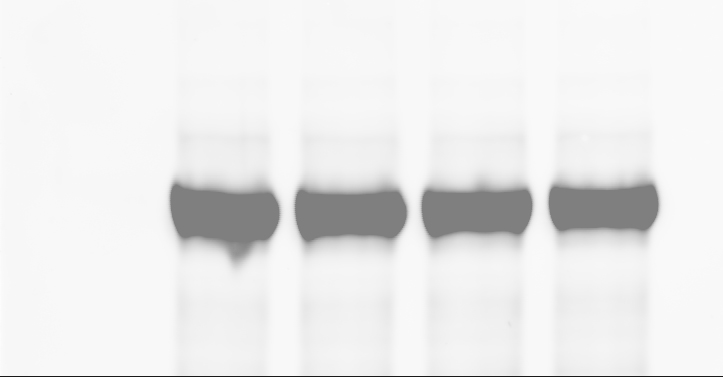

Supplement: Figure 2—source data 1. [file elife-88637-fig2-data1.zip › Figure2-source data1/Figure2E/Figure2E-5.jpg]

Figure2A

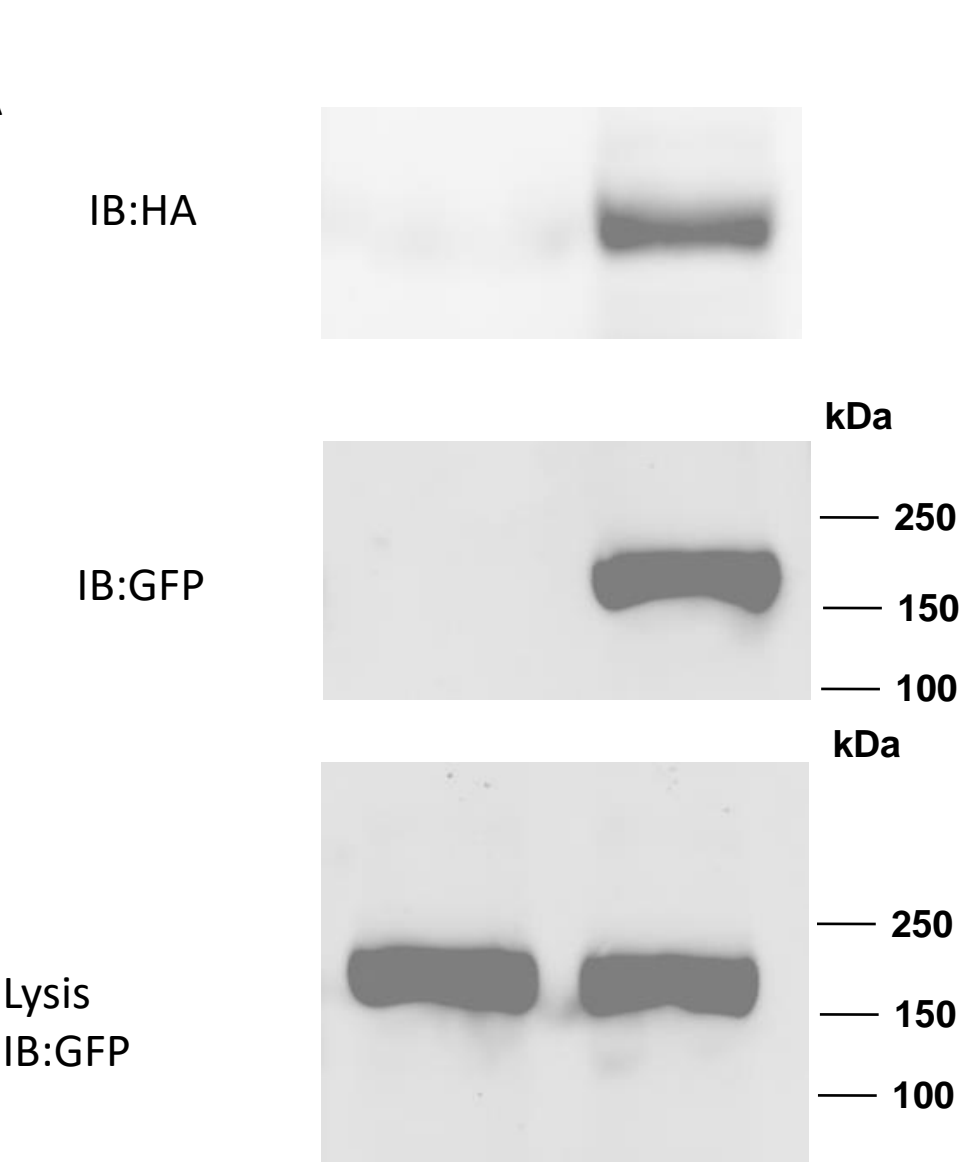

Figure2B

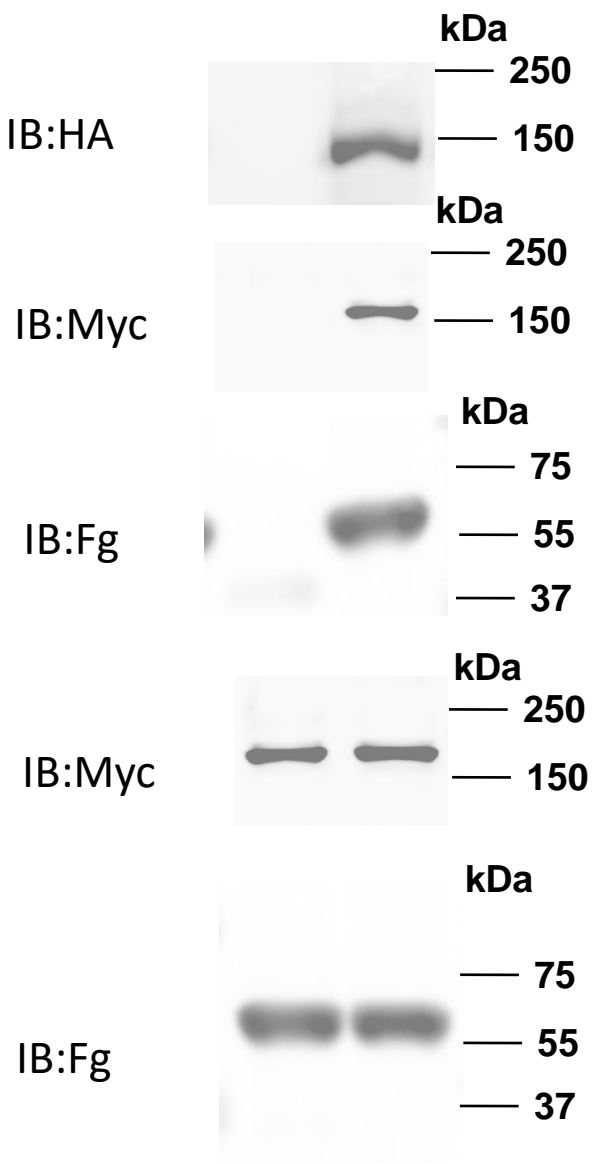

Figure2D

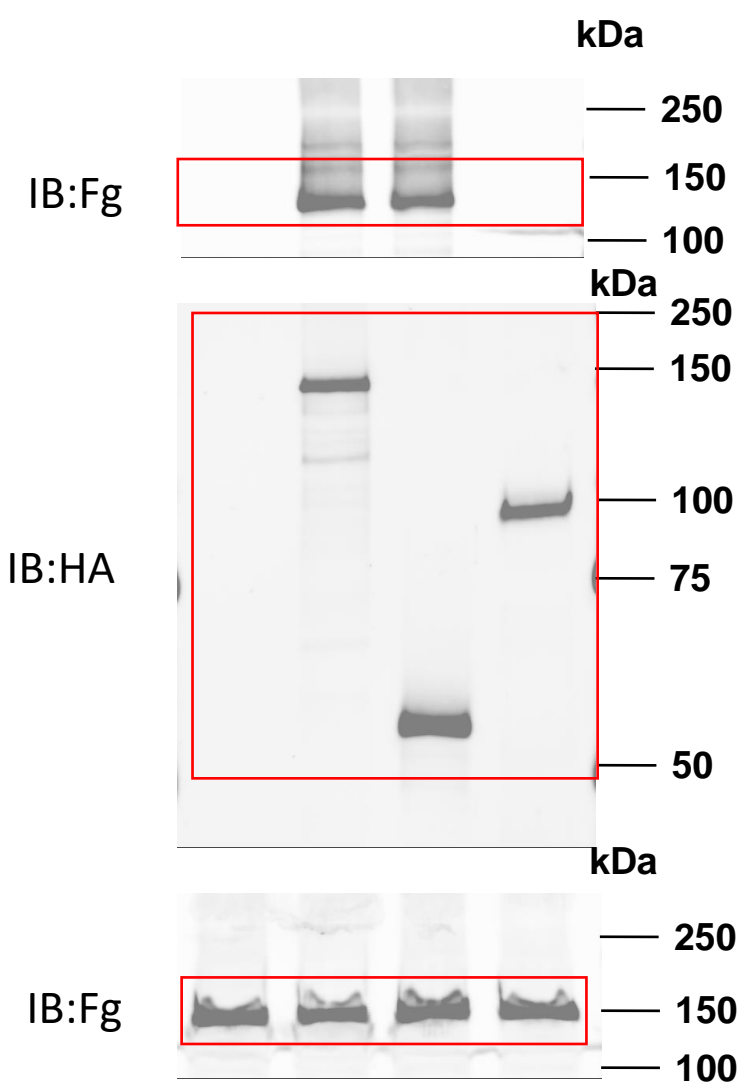

Figure2E

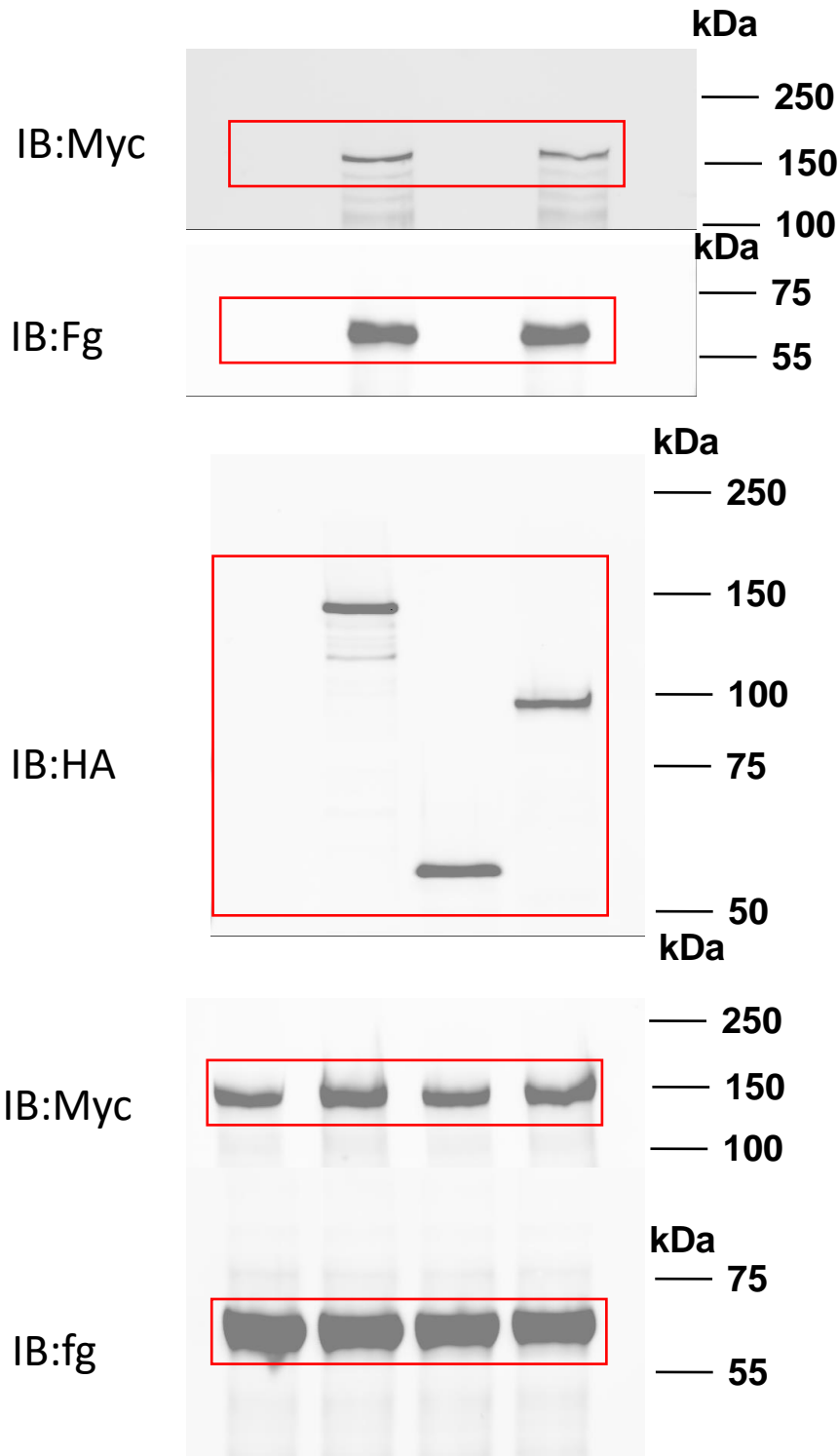

Supplement: Figure 2—source data 1. [file elife-88637-fig2-data1.zip › Figure2-source data1/Figure2-source data 1.pdf]

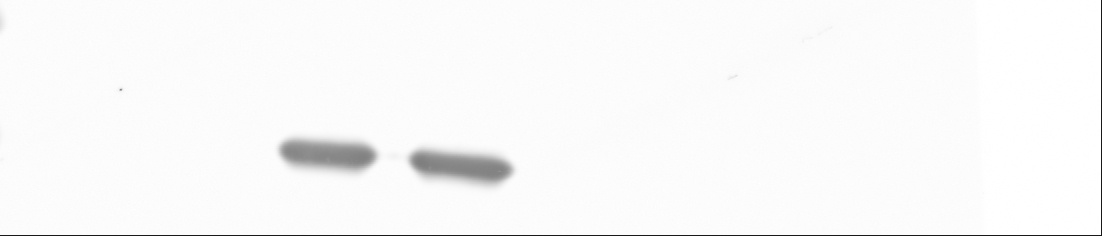

Supplement: Figure 3—source data 1. [file elife-88637-fig3-data1.zip › Figure3-source data3/Figure3A/Figure3A-1.jpg]

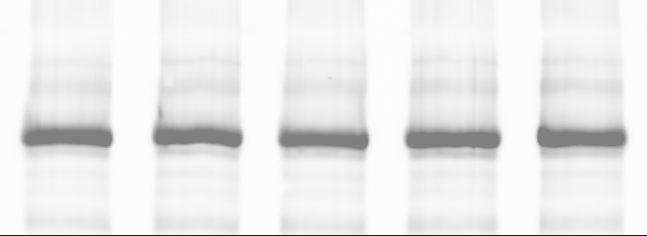

Supplement: Figure 3—source data 1. [file elife-88637-fig3-data1.zip › Figure3-source data3/Figure3A/Figure3A-2.jpg]

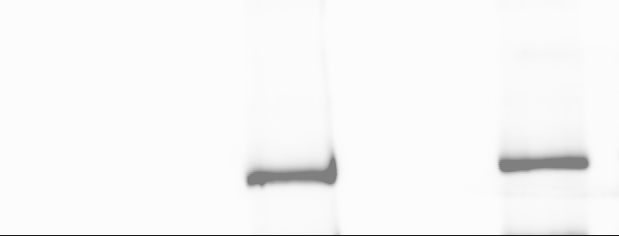

Supplement: Figure 3—source data 1. [file elife-88637-fig3-data1.zip › Figure3-source data3/Figure3A/Figure3A-3.jpg]

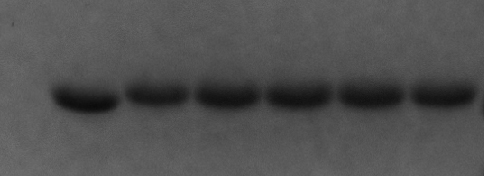

Supplement: Figure 3—source data 1. [file elife-88637-fig3-data1.zip › Figure3-source data3/Figure3A/Figure3A-4.jpg]

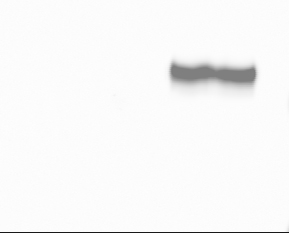

Supplement: Figure 3—source data 1. [file elife-88637-fig3-data1.zip › Figure3-source data3/Figure3C/Figure3C-1.jpg]

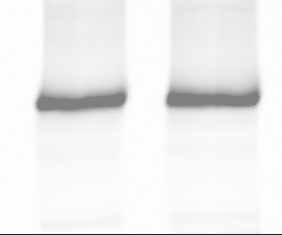

Supplement: Figure 3—source data 1. [file elife-88637-fig3-data1.zip › Figure3-source data3/Figure3C/Figure3C-2.jpg]

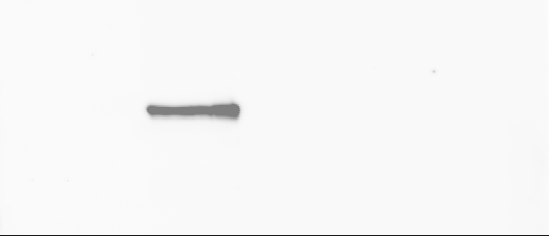

Supplement: Figure 3—source data 1. [file elife-88637-fig3-data1.zip › Figure3-source data3/Figure3D/Figure3D-1.jpg]

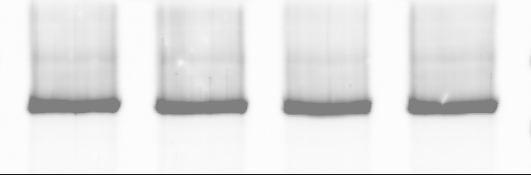

Supplement: Figure 3—source data 1. [file elife-88637-fig3-data1.zip › Figure3-source data3/Figure3D/Figure3D-2.jpg]

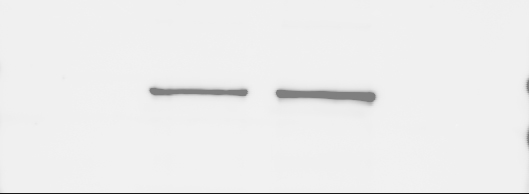

Supplement: Figure 3—source data 1. [file elife-88637-fig3-data1.zip › Figure3-source data3/Figure3E/Figure3E-1.jpg]

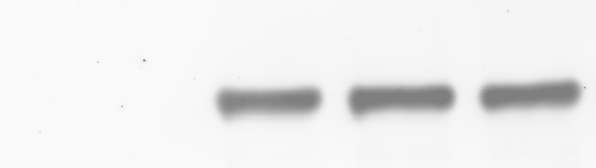

Supplement: Figure 3—source data 1. [file elife-88637-fig3-data1.zip › Figure3-source data3/Figure3E/Figure3E-2.jpg]

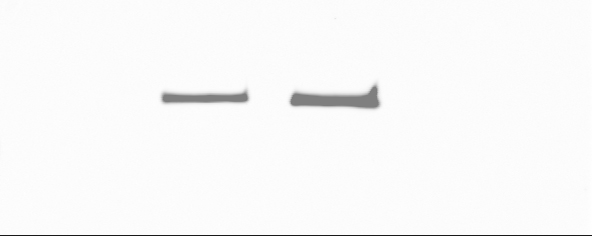

Supplement: Figure 3—source data 1. [file elife-88637-fig3-data1.zip › Figure3-source data3/Figure3G/Figure3G-1.jpg]

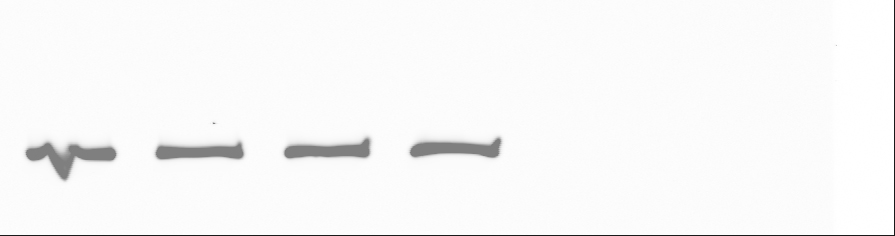

Supplement: Figure 3—source data 1. [file elife-88637-fig3-data1.zip › Figure3-source data3/Figure3G/Figure3G-2.jpg]

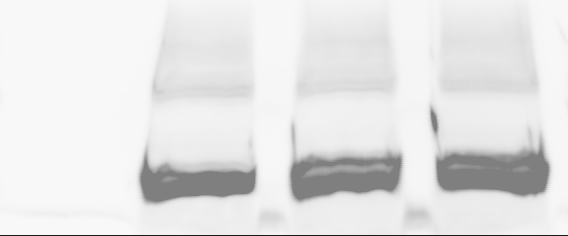

Supplement: Figure 3—source data 1. [file elife-88637-fig3-data1.zip › Figure3-source data3/Figure3G/Figure3G-3.jpg]

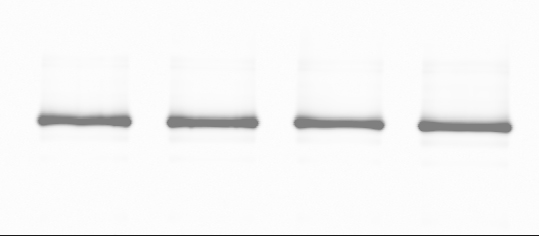

Supplement: Figure 3—source data 1. [file elife-88637-fig3-data1.zip › Figure3-source data3/Figure3I/Figure3I-1.jpg]

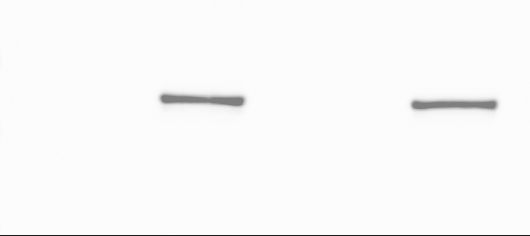

Supplement: Figure 3—source data 1. [file elife-88637-fig3-data1.zip › Figure3-source data3/Figure3I/Figure3I-2.jpg]

Figure3A

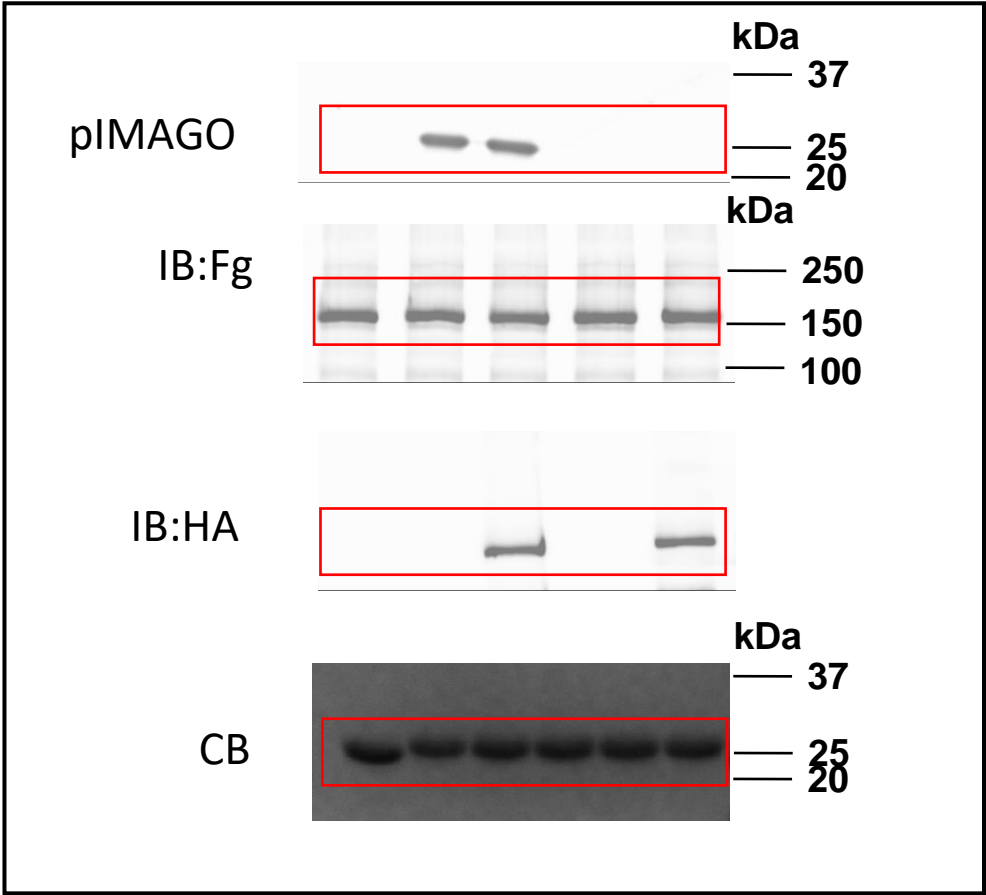

Figure3C

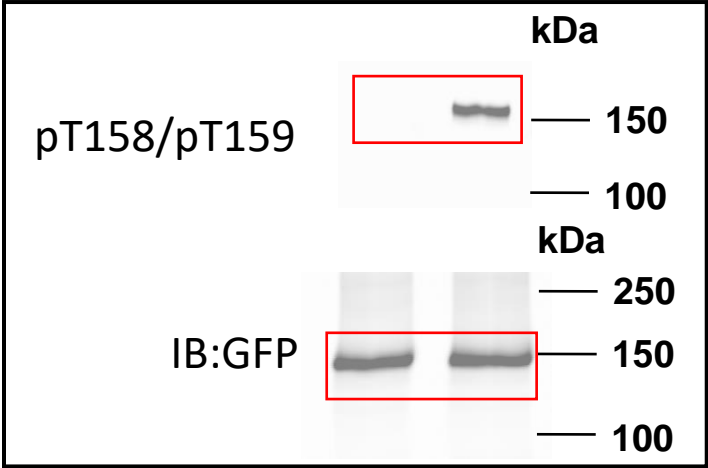

Figure3D

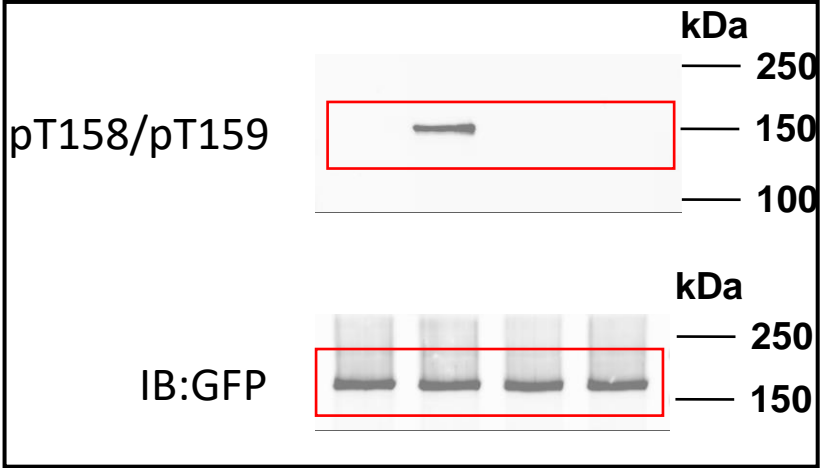

Figure3E

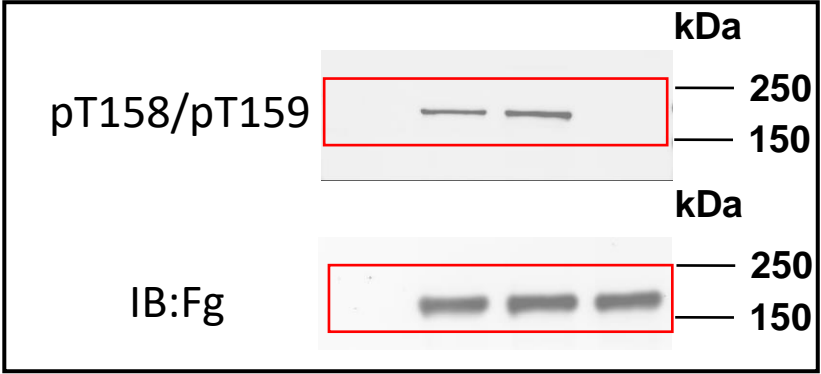

Figure3I

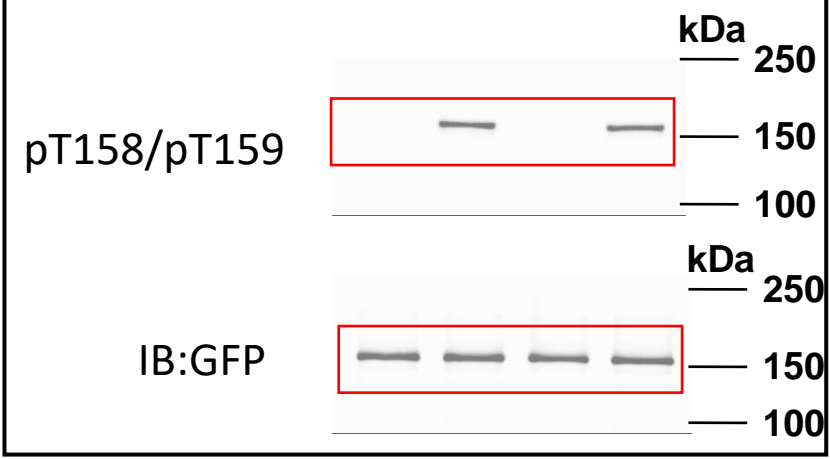

Figure3G

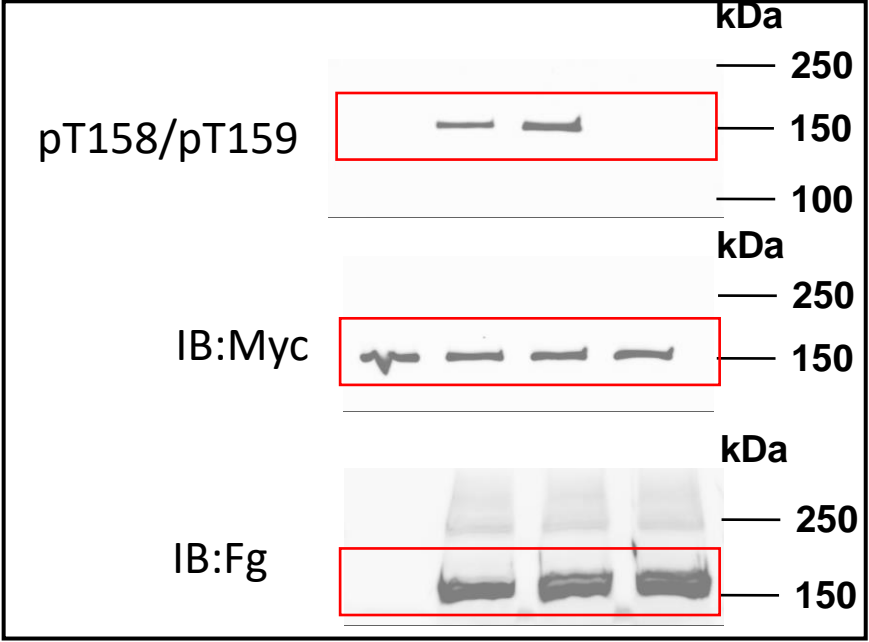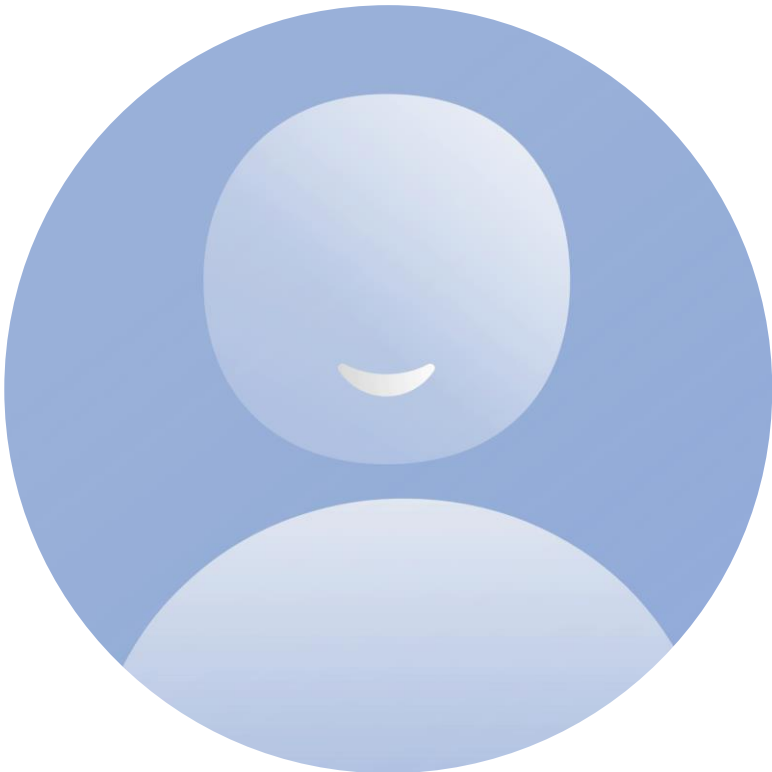

Supplement: Figure 3—source data 1. [file elife-88637-fig3-data1.zip › Figure3-source data3/Figure3-source data 1.pdf]

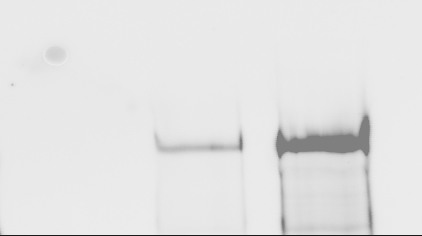

Supplement: Figure 4—source data 1. [file elife-88637-fig4-data1.zip › Figure4-source data1/Figure4A/Figure4A-1.jpg]

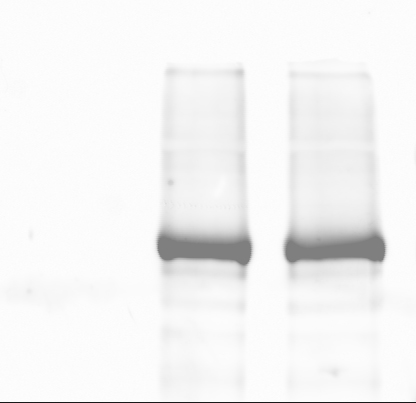

Supplement: Figure 4—source data 1. [file elife-88637-fig4-data1.zip › Figure4-source data1/Figure4A/Figure4A-2.jpg]

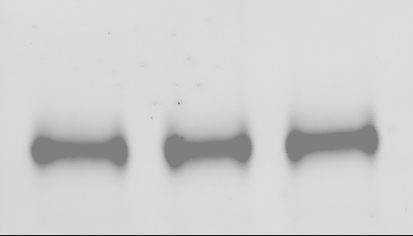

Supplement: Figure 4—source data 1. [file elife-88637-fig4-data1.zip › Figure4-source data1/Figure4A/Figure4A-3.jpg]

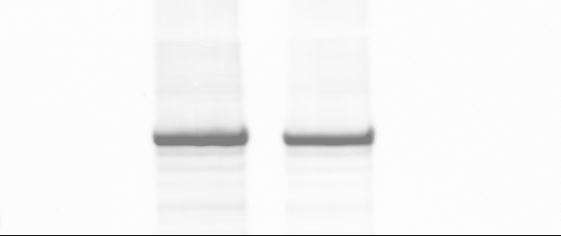

Supplement: Figure 4—source data 1. [file elife-88637-fig4-data1.zip › Figure4-source data1/Figure4C/Figure4C-1.jpg]

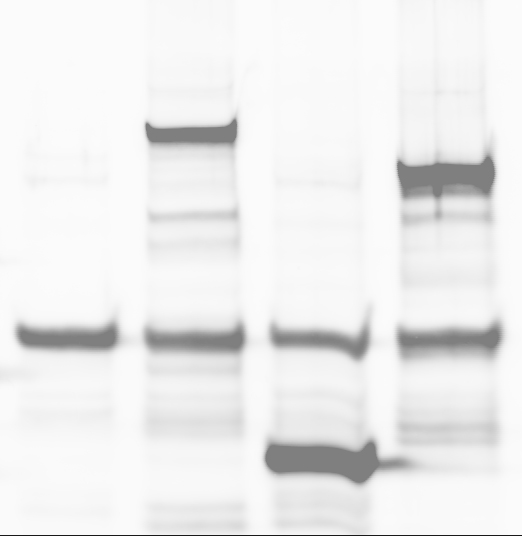

Supplement: Figure 4—source data 1. [file elife-88637-fig4-data1.zip › Figure4-source data1/Figure4C/Figure4C-2.jpg]

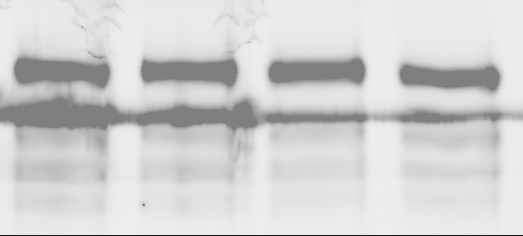

Supplement: Figure 4—source data 1. [file elife-88637-fig4-data1.zip › Figure4-source data1/Figure4C/Figure4C-3.jpg]

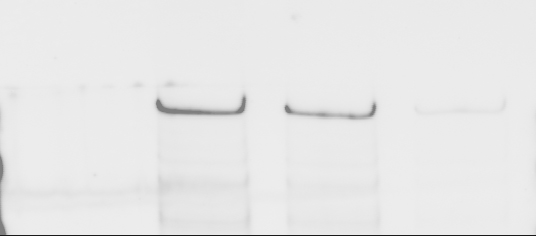

Supplement: Figure 4—source data 1. [file elife-88637-fig4-data1.zip › Figure4-source data1/Figure4D/Figure4D-1.jpg]

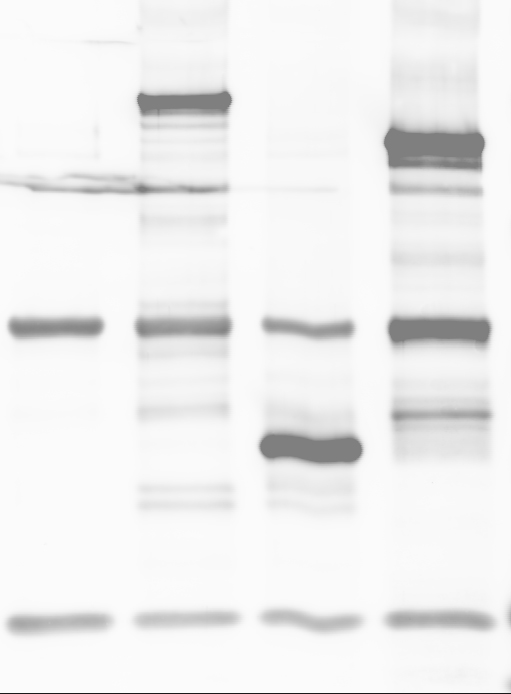

Supplement: Figure 4—source data 1. [file elife-88637-fig4-data1.zip › Figure4-source data1/Figure4D/Figure4D-2.jpg]

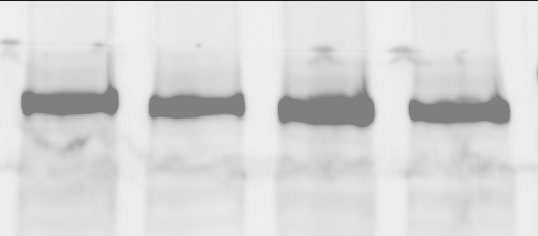

Supplement: Figure 4—source data 1. [file elife-88637-fig4-data1.zip › Figure4-source data1/Figure4D/Figure4D-3.jpg]

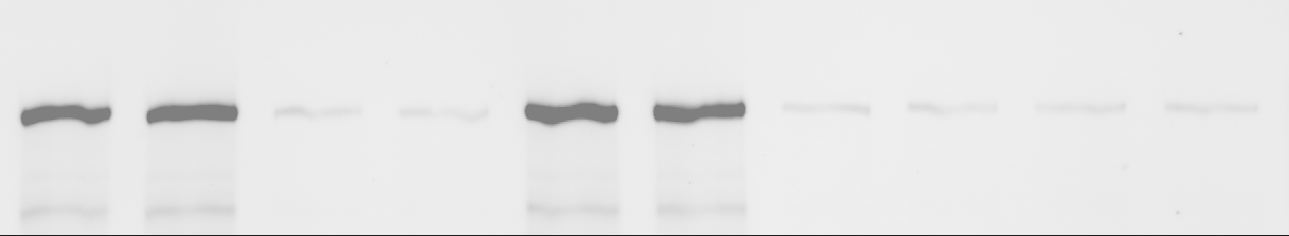

Supplement: Figure 4—source data 1. [file elife-88637-fig4-data1.zip › Figure4-source data1/Figure4E/Figure4E-1.jpg]

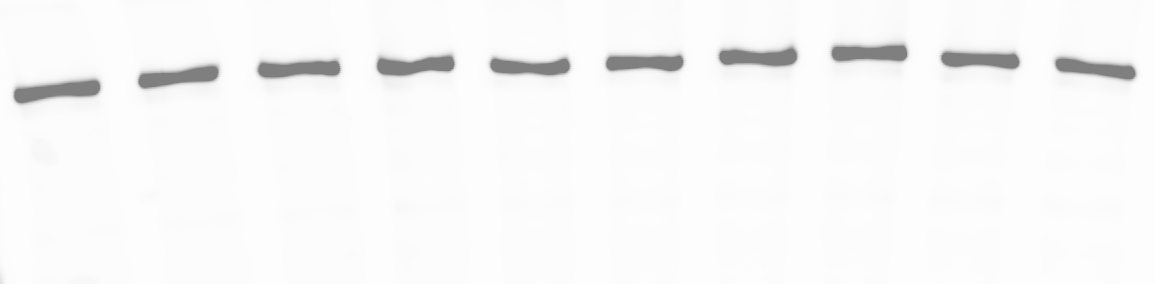

Supplement: Figure 4—source data 1. [file elife-88637-fig4-data1.zip › Figure4-source data1/Figure4E/Figure4E-2.jpg]

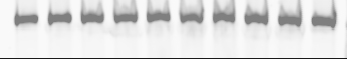

Supplement: Figure 4—source data 1. [file elife-88637-fig4-data1.zip › Figure4-source data1/Figure4E/Figure4E-3.jpg]

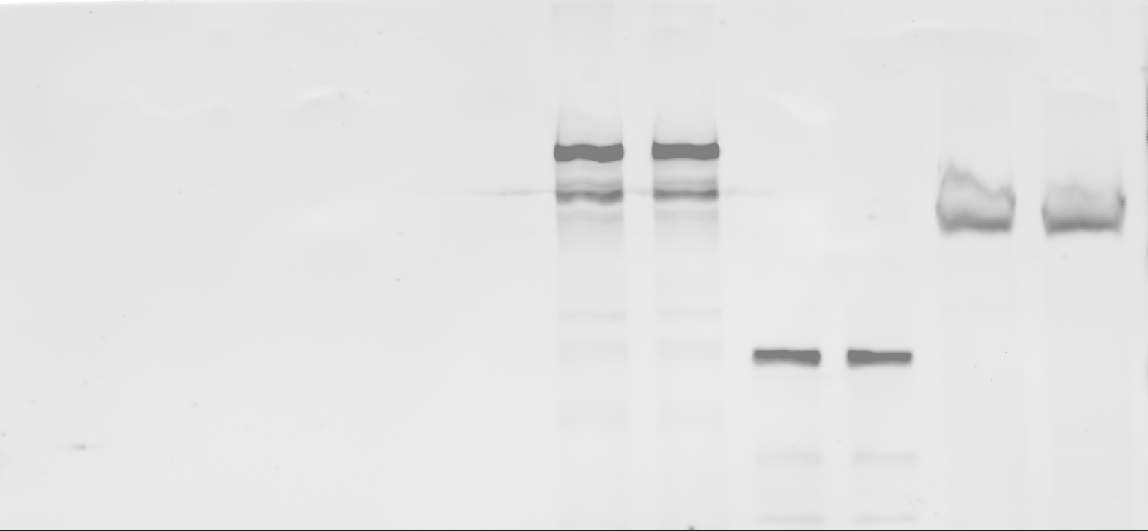

Supplement: Figure 4—source data 1. [file elife-88637-fig4-data1.zip › Figure4-source data1/Figure4E/Figure4E-4.jpg]

Figure4A

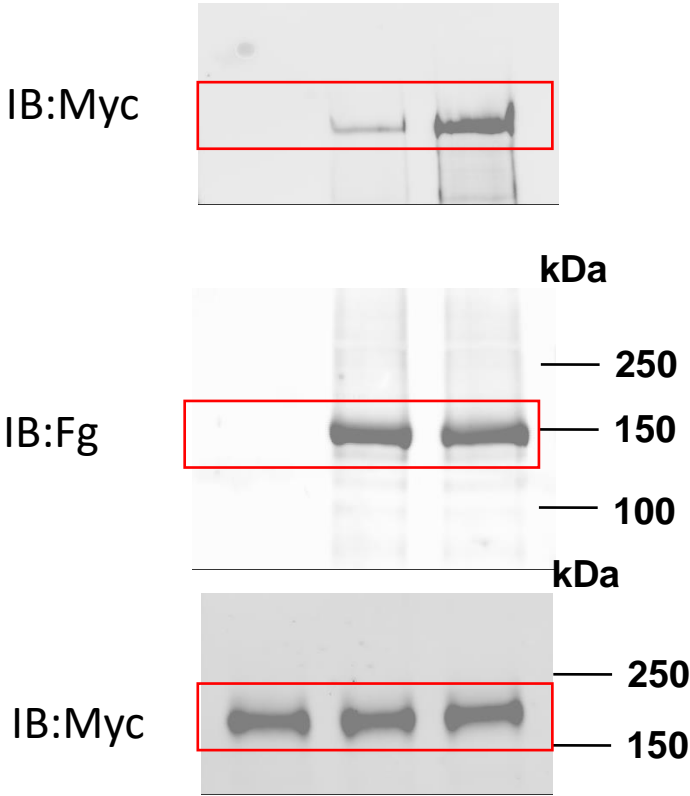

Figure4C

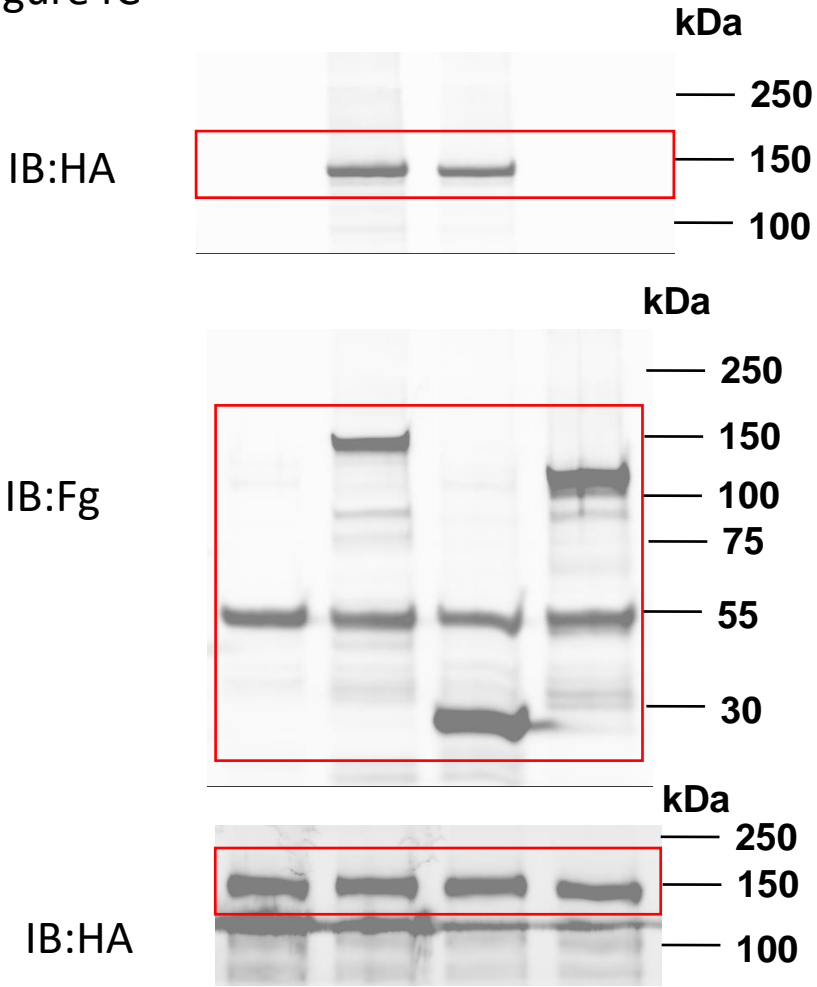

Figure4D

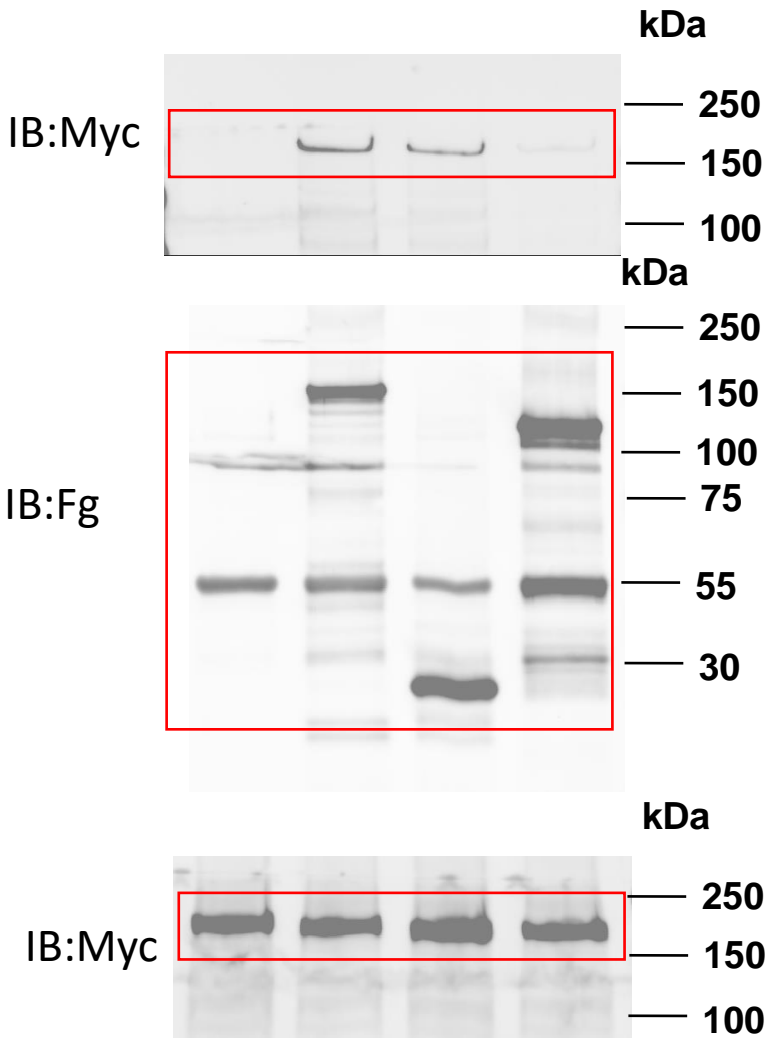

Figure4E

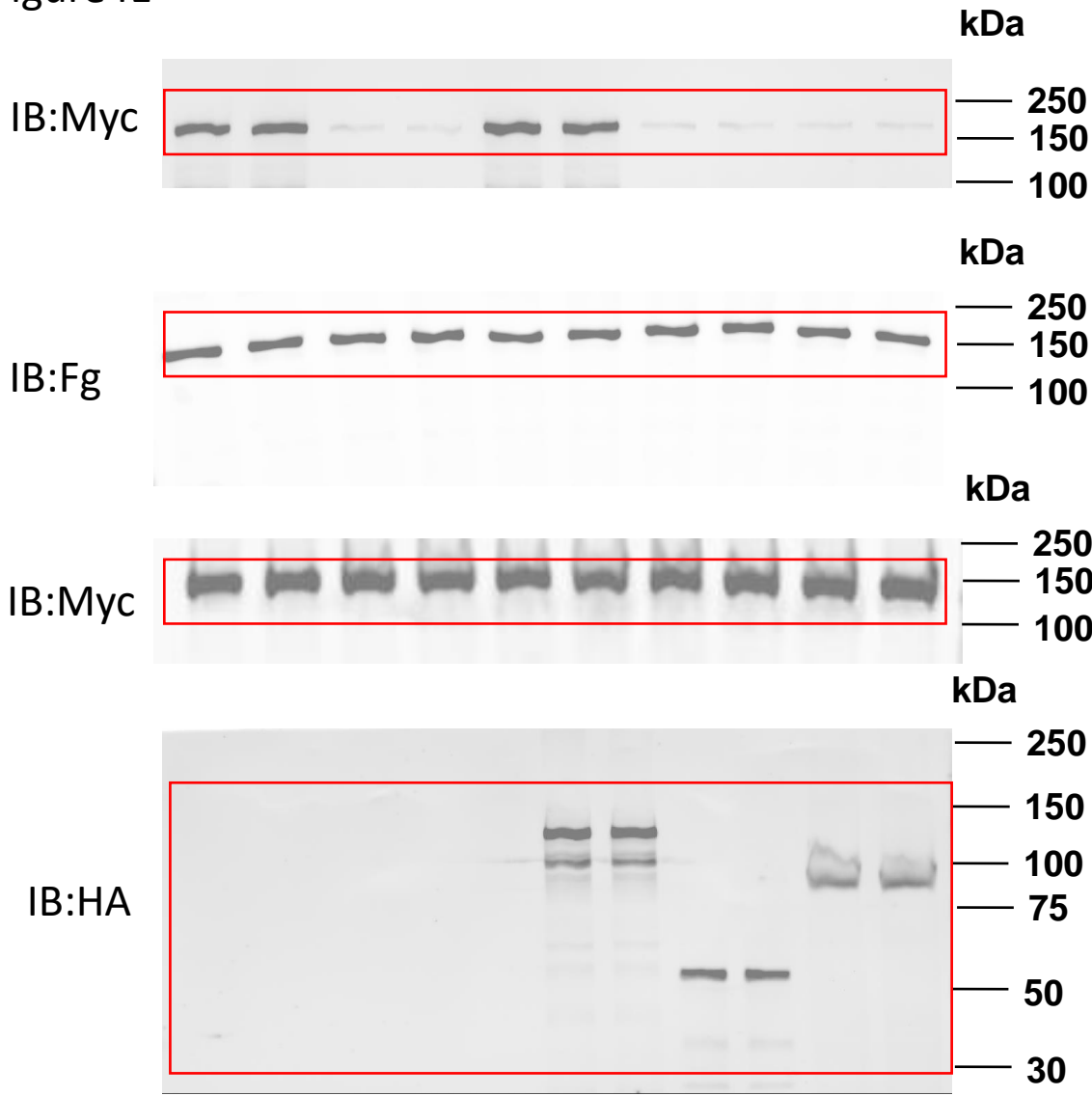

Supplement: Figure 4—source data 1. [file elife-88637-fig4-data1.zip › Figure4-source data1/Figure4-source data 1.pdf]

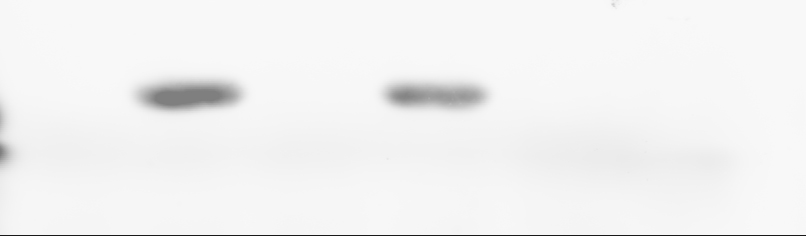

Supplement: Figure 7—source data 1. [file elife-88637-fig7-data1.zip › Figure7-source data1/Figure7G-1.jpg]

Figure7G

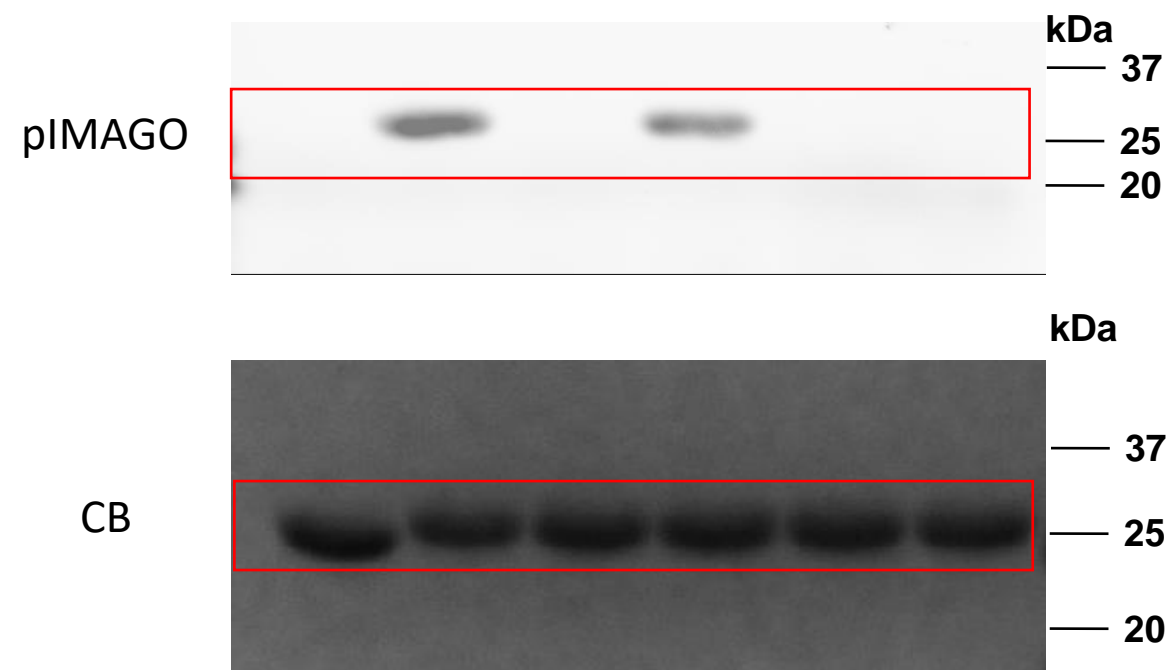

Supplement: Figure 7—source data 1. [file elife-88637-fig7-data1.zip › Figure7-source data1/Figure7-source data 1.pdf]

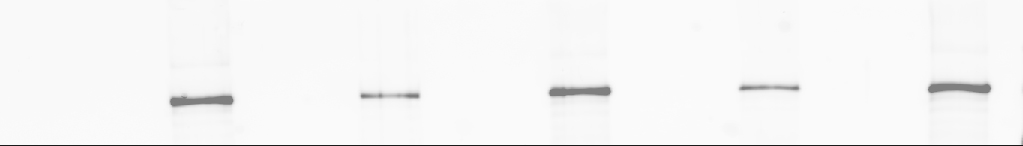

Supplement: Figure 8—source data 1. [file elife-88637-fig8-data1.zip › Figure8-source data1/Figure8C-1.jpg]

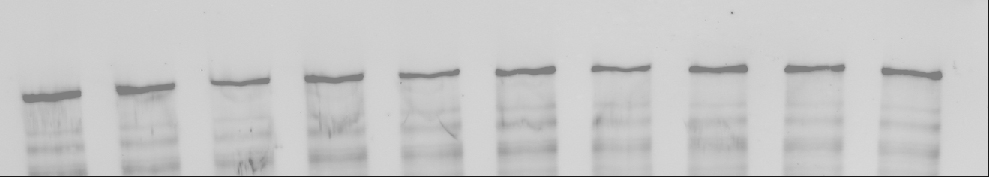

Supplement: Figure 8—source data 1. [file elife-88637-fig8-data1.zip › Figure8-source data1/Figure8C-2.jpg]

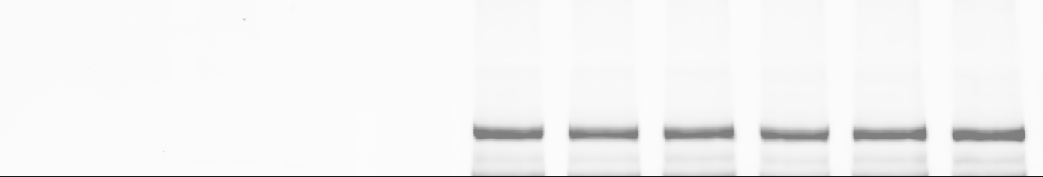

Supplement: Figure 8—source data 1. [file elife-88637-fig8-data1.zip › Figure8-source data1/Figure8C-3.jpg]

Figure8C

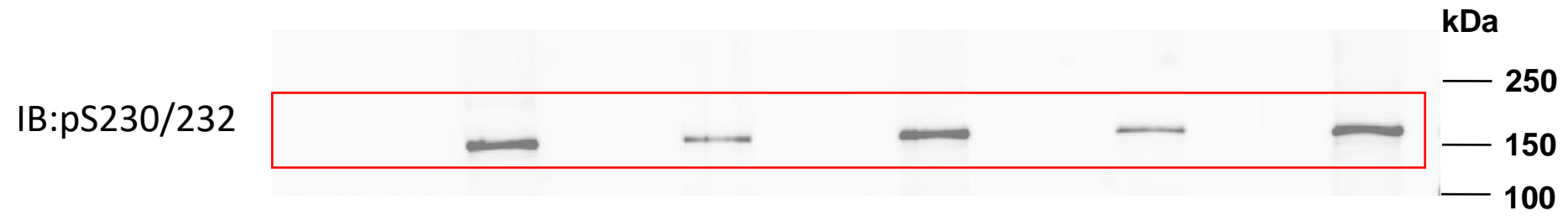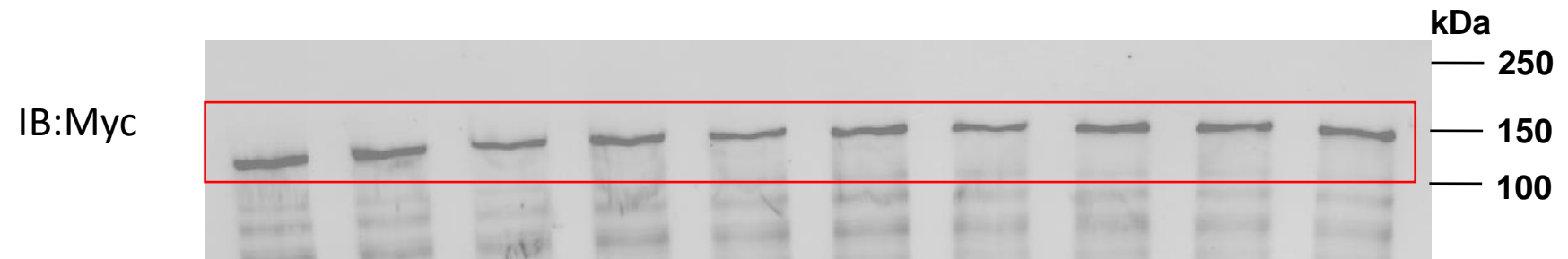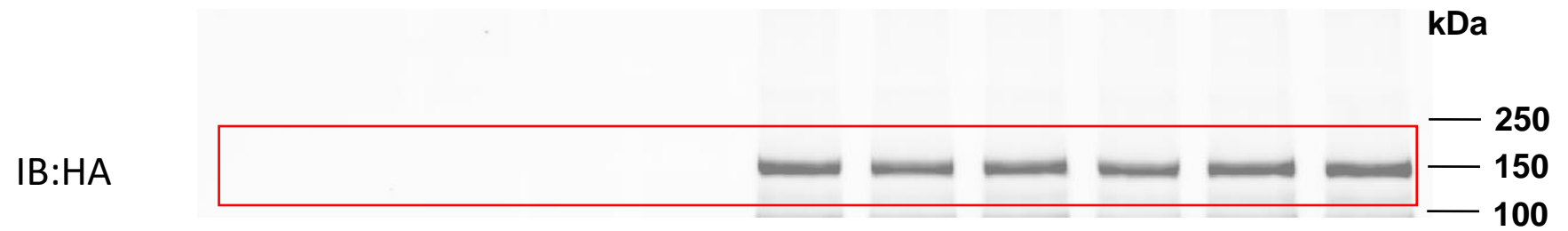

Supplement: Figure 8—source data 1. [file elife-88637-fig8-data1.zip › Figure8-source data1/Figure8-source data 1.pdf]
