# Supplementary material for: Ulk4 promotes Shh signaling by regulating Stk36 ciliary localization and Gli2 phosphorylation
Source: eLife. 2023 Dec 14;12:RP88637. doi: 10.7554/eLife.88637 (PMC10721220; doi:10.7554/eLife.88637)
Supplement: Figure 8—source data 1. [file elife-88637-fig8-data1.zip › Figure8-source data1/Figure8-source data 1.docx]

**Figure 8—source data 1**

Uncropped Western Blots as well as immunoblots including sample and band identification are provided for data presented in Figure 8.
